# Menstrual Cycle and Progesterone as Future Treatment Targets of Alcohol Use Disorder

**DOI:** 10.1192/j.eurpsy.2025.124

**Published:** 2025-08-26

**Authors:** B. Lenz, ReCoDe Consortium

**Affiliations:** Addictive Behavior and Addiction Medicine, Central Institute of Mental Health, Mannheim, Germany

## Abstract

**Abstract:**

Alcohol use disorder (AUD) has a significant impact on the individuals affected, their relatives, and the society at large. Given the limited efficacy of currently available treatments, there is a pressing need for a more comprehensive mechanistic understanding. In particular, consideration must be given to the dynamical and real-world aspects. The objective of this study was to investigate the association of dynamic variations across menstrual cycle phases (indicative of progesterone-to-estradiol ratios) in women with AUD and progesterone-to-estradiol ratios in men with AUD with real-world problem drinking. To this end, longitudinal data from the German TRR265 cohort (comprising smartphone entries on alcohol consumption, craving, and loss of control, self-reports on menstrual cycle phases, and blood progesterone-to-estradiol ratios) were subjected to analysis. In women with AUD, the lowest levels of problem drinking, craving, and loss of control were observed during the late luteal phase, when the progesterone-to-estradiol ratios reached their peak. Similarly, in men with AUD, higher progesterone-to-estradiol ratios were associated with lower problem drinking, craving, and loss of control. Some of these effects were moderated by the severity of the AUD. The results highlight the progesterone-to-estradiol ratio as a promising future treatment target and point to the necessity of cycle phase-tailored treatments for women with AUD.

**Disclosure of Interest:**

None Declared

